# Effect of dry dynamic apnea on aerobic power in elite rugby athletes: a warm-up method

**DOI:** 10.3389/fphys.2023.1269656

**Published:** 2024-01-16

**Authors:** Wang Wendi, Wu Dongzhe, Wang Hao, Shi Yongjin, Gao Xiaolin

**Affiliations:** ^1^ Sports Rehabilitation Research Center, China Institute of Sport Science, Beijing, China; ^2^ Department of Sports and Arts, China Agricultural University, Beijing, China

**Keywords:** dynamic apnea, breath-holding, diving reflex, elite athletes, cardiorespiratory optimal point

## Abstract

**Objective:** While long-term dynamic breath-holding training has been extensively studied to enhance cardiopulmonary function in athletes, limited research has explored the impact of a single breath-holding session on subsequent athletic capacity. In addition, Dry Dynamic Apnea (DA) has a more immediate physiological response than wet and static breath-holding. This study aims to assess the immediate effects of a single session of DA on the aerobic power and hematological parameters of elite athletes.

**Methods:** Seventeen elite male rugby athletes (average age 23.5 ± 1.8) participated in this study. Two warm-up protocols were employed prior to incremental exercise: a standard warm-up (10 min of no-load pedaling) and a DA warm-up (10 min of no-load pedaling accompanied by six maximum capacity breath holds, with 30 s between each breath hold). Fingertip blood indicators were measured before and after warm-up. The incremental exercise test assessed aerobic parameters with self-regulation applied throughout the study.

**Results:** Compared to the baseline warm-up, the DA warm-up resulted in a significant increase in VO2peak from 3.14 to 3.38 L/min (7.64% change, *p* < 0.05). HRmax increased from 170 to 183 bpm (7.34% change, *p* < 0.05), and HRpeak increased from 169 to 182 bpm (7.52% change, *p* < 0.05). Hematocrit and hemoglobin showed differential changes between the two warm-up methods (P_Hematocrit_ = 0.674; P_hemoglobin_ = 0.707).

**Conclusion:** This study investigates how DA influences physiological factors such as spleen contraction, oxygen uptake, and sympathetic nerve activation compared to traditional warm-up methods. Immediate improvements in aerobic power suggest reduced vagus nerve stimulation, heightened sympathetic activity, and alterations in respiratory metabolism induced by the voluntarily hypoxia-triggered warm-up. Further research is warranted to comprehensively understand these physiological responses and optimize warm-up strategies for elite athletic performance.

## 1 Introduction

Apnea, or breath-holding, stands as a widely acknowledged method that instigates diverse physiological responses, with the prominent diving reflex being a notable example (de Asís-Fernández et al., 2022; Fitz-Clarke, 2018). This response encompasses bradycardia, spleen contraction, and heightened peripheral vascular resistance (Eichhorn et al., 2017). In apnea disciplines, performance hinges on three key physiological factors: overall gas storage capacity (for oxygen and carbon dioxide buffering) (Stroud, 1959), tolerance to asphyxia determining blackout threshold, and metabolic rate inversely correlated with apnea duration (Mithoefer, 1959b). Engaging in activities that increase oxygen consumption or lactate levels just before a dive is counterproductive (Lin et al., 1974). It serves as the cornerstone of training for breath-hold divers (Perini et al., 2008), showcasing remarkable advancements in oxygen storage and exchange capabilities (Asmussen and Kristiansson, 1968; Bain et al., 2018; Msg, 2023). Notably, a singular spleen contraction elevates the overall oxygen content of blood by 2.8%–9.6% (Bergman et al., 1972), and its induction is more readily achieved during less intense exercise (Andersson and Evaggelidis, 2009), a pattern akin to the conditioning effect observed during warm-up.

Dry Dynamic Apnea (DA), as a distinctive technique within breath-holding training, sets itself apart from other methods. It requires individuals to hold their breath while simultaneously engaging in regular leg movements (Trincat et al., 2017; de Asís-Fernández et al., 2019; Bouten et al., 2022; Godek and Freeman, 2022). In comparison to static or wet methods, dry breath-hold warm-up induces a more pronounced diving response, avoiding elevated lactate levels, and demonstrating a stronger physiological response (Hwang et al., 2006; Vitali et al., 2022). On one hand, DA serves as a stimulus for parasympathetic activation, effectively increasing vagus tension and reducing unnecessary oxygen consumption (Ferretti and Costa, 2003). On the other hand, exercise itself leads to a heightened heart rate (HR), vasodilation of peripheral skeletal muscles, and the redistribution of blood flow to supply essential organs with oxygen (Heistad et al., 1968; Leuenberger et al., 2001). This may initially appear paradoxical, but the utilization of DA in the warm-up phase can mitigate energy loss, resulting in subsequent energy savings. Moreover, DA improves lung ventilation, blood oxygen transport, and peripheral oxygen utilization during exercise, all of which are crucial factors affecting cardiorespiratory endurance (Lemaître et al., 2009; Woorons et al., 2008; Schagatay, 2010; Elia et al., 2021a). DA combines breath holding and warm-up, does not require subjects to immerse their faces in water, and seems to be more convenient and effective in producing acute physiological effects on subsequent exercise.

While traditionally VO2peak is a valuable metric for assessing aerobic power, relying on a single indicator may not provide a comprehensive evaluation. Sports scientists and coaches, when conducting incremental exercise tests for performance diagnostics, should comprehensively consider the impact of multiple indicators, including the ventilatory threshold, on endurance performance or sensitivity (Bentley et al., 2007). In 2012, the Ramos team introduced the concept of the “Cardiorespiratory Optimal Point” (COP) after discovering a distinctive “U”-shaped pattern in the human body’s oxygen uptake curve during incremental load exercise (Ramos et al., 2014). This U-shaped curve marks an optimal efficiency point for oxygen utilization during transitions from rest to exercise and from exercise to recovery. COP, a reproducible and physiologically based variable in CPET, is linked to all-cause mortality in the general population during routine clinical exercise assessments (Laukkanen, 2018). Specifically, COP is defined as the lowest VE/VO2 ratio within a minute during CPET (Ramos et al., 2012), often aligning with the first respiratory threshold (Ramos and Araújo, 2017; Silva et al., 2018). Considering athletes’ limited opportunities for repeated maximal CPET sessions during the season, COP becomes a more feasible and acceptable variable to measure and monitor consistently (Christina et al., 2018). Additionally, a study by Castro et al. (2018), focusing on high-level football players undergoing CPET on a treadmill, revealed consistent COP values across different field positions, suggesting its potential utility among elite athletes (Laukkanen et al., 2021). In a cross-sectional study on students’ cardiorespiratory fitness, our research team employed a back-propagation neural network model based on the COP to accurately predict VO2peak.

We propose that DA, identified as ‘voluntarily hypoxia-triggered training,’ holds the potential to enhance aerobic power (Elia et al., 2021b). The primary objective of this study is to assess the immediate impact of DA on both aerobic performance indicators and hematological parameters. Our hypothesis revolves around two key aspects: Firstly, we anticipate that DA will promptly induce changes in aerobic performance, particularly in elite athletes. Secondly, we predict that DA will result in more significant fluctuations in hematological parameters, specifically in Red Blood Cell count (RBC) and hemoglobin (HGB), compared to traditional dynamic warm-up methods.

## 2 Materials and methods

### 2.1 Ethical approval

The entire protocol received approval from the Ethics Committee of the China Institute of Sports Science. Written informed consent was obtained from all subjects after a comprehensive explanation of the test procedures and potential risks. Before the formal trial, participants were informed of the comprehensive trial procedure and its purpose.

### 2.2 Population

Sample size calculation was conducted using G*Power 3.1 software, confirming the sufficiency of the sample size for this study. Statistical power analysis indicates that, with a significance level (α) of 0.05, a medium effect size (Cohen’s d = 0.65), and a statistical power of ≥0.7, a total of 17 participants are required for the survey.

We recruited 21 elite male rugby athletes through online means at China Agricultural University, following random selection. Exclusion criteria included the following: (1) Had a presence of cardiac, cerebral, and vascular disease or a family history of sudden death; (2) Motor impairment of the extremities within the past 6 months; (3) History of smoking or drinking; (4) Individuals with any history of respiratory training, including but not limited to practices such as yoga, meditation, or other related respiratory interventions within the past 6 months, will be excluded. All subjects who completed the trial were considered valid.

Seventeen individuals accepted and completed the trial, while four individuals were deemed invalid due to personal circumstances. General characteristics can be seen in Table 1.

**TABLE 1:** General characteristics (X ± S).

General characteristics	
Age (years)	23.5 ± 1.8
Height (cm)	182.2 ± 4.6
Body mass (kg)	83.8 ± 8.5
BMI (kg/m^2^)	25.2 ± 1.8

### 2.3 Procedures

The trial was conducted in May 2023 at the Sports Cardio Exercise Laboratory of the China Institute of Sports Science. The study spanned two separate test days. On the initial test day, we measured essential information and conducted baseline testing. The baseline test involved incremental exercise, which included a baseline warm-up with normal respiration. On the second test day, the subjects engaged in incremental exercise, which included a warm-up involving DA. Subjects performed 5 min of lower limb muscle stretching as a basic warm-up before both tests to prevent exercise damage.

**FIGURE 1:**
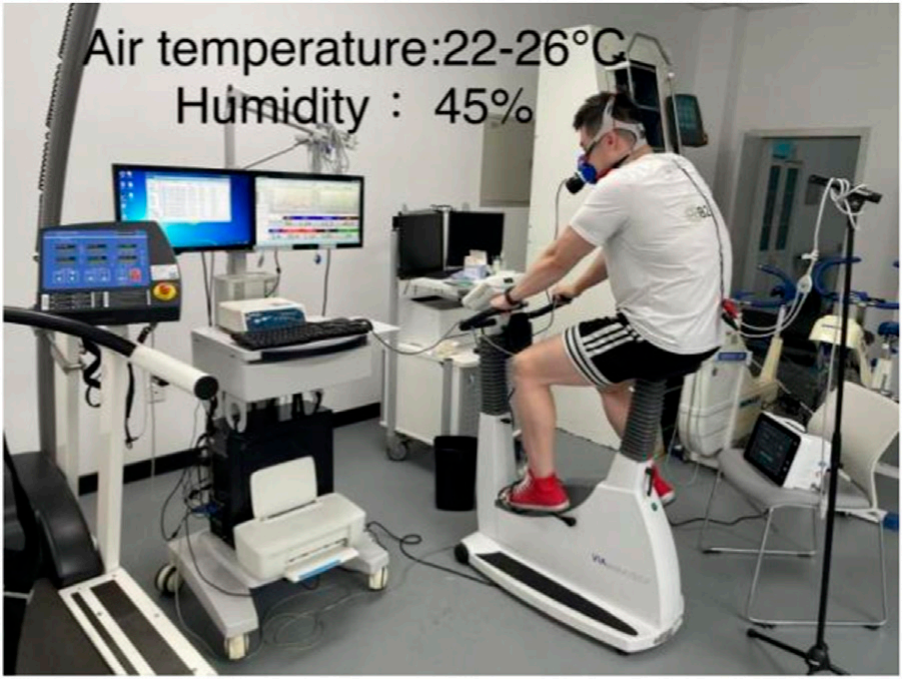
Example of the experimental setup.

This study utilized the random number generation method of the SPSS software to determine, in a randomized fashion, the order in which subjects participated in the baseline group or DA warm-up group test (baseline first week or DA first week). To minimize potential residual effects on the first testing day, a physiological washout period of 1 week was implemented between baseline and DA trials. To account for possible circadian rhythm effects, all subjects were tested between 9:30 and 11:00 a.m. to ensure that both experiments on the same subject were conducted during the same time period. Participants were instructed not to consume caffeine or alcoholic beverages within the 48 h preceding the test day. Figure 1 is an example of the experimental setup.

### 2.4 Incremental exercise

The experiment was carried out in a cardiopulmonary exercise laboratory with a temperature of 22°C–26°C and a humidity of 45%. Subjects were required to remain awake and at rest for 30 min before commencing the test. Testers then assisted subjects in positioning themselves on an upright power bike, adjusted seat height, and fitted them with a wireless telemetry exercise cardiopulmonary tester. All exercise tests were carried out using an electrically vertical power bike (Eegoselect 100, Ergoline Academy, Germany). A wireless telemetric exercise cardiopulmonary tester (Cortex MetaMax 3B, Germany) was utilized to measure cardiopulmonary motor function indicators. Different types of masks were made available for selection based on the subjects’ preferences.

Based on the previous research experience of our team, the experimental procedure is divided into the following stages:

1. Quiet Phase: 5 min of sitting at rest.
2. First Blood Collection: At the end of the Quiet Phase, collected peripheral blood from the fingertip.
3. Warm-up Phase: The baseline group pedals at 0 W/min for 10 min. The dynamic breath-holding group, on the other hand, also pedals at 0 W/min for 10 min and performs six maximal breath-holding maneuvers with a 30-s interval between each. Participants can end the breath-holding when the desire to breathe again surpasses their willingness to continue.
4. Second Blood Collection: At the end of the Warm-up Phase, peripheral blood from the fingertip. The punctures are not repeated at the first site (two separate punctures).
5. Exercise Phase: The exercise protocol commenced with an initial load of 40 W, sustained for a duration of 2 min. Subsequently, a linear load increment of 25 W/min was applied.

Reaching 90% of the individual’s maximum predicted heart rate (calculated as 210 - (age × 0.65)) (Dane, 1944; Achten and Jeukendrup, 2003; Society, 2003; Gelbart et al., 2017); Rate of Perceived Exertion (RPE) ≥ 17); Decline in oxygen uptake during sustained exercise changes or a decrease in oxygen uptake during continuous exercise was considered. Meeting any one of these criteria could be regarded as reaching the maximum load (Balady et al., 2010; Zimmermann et al., 2022).

**FIGURE 2:**
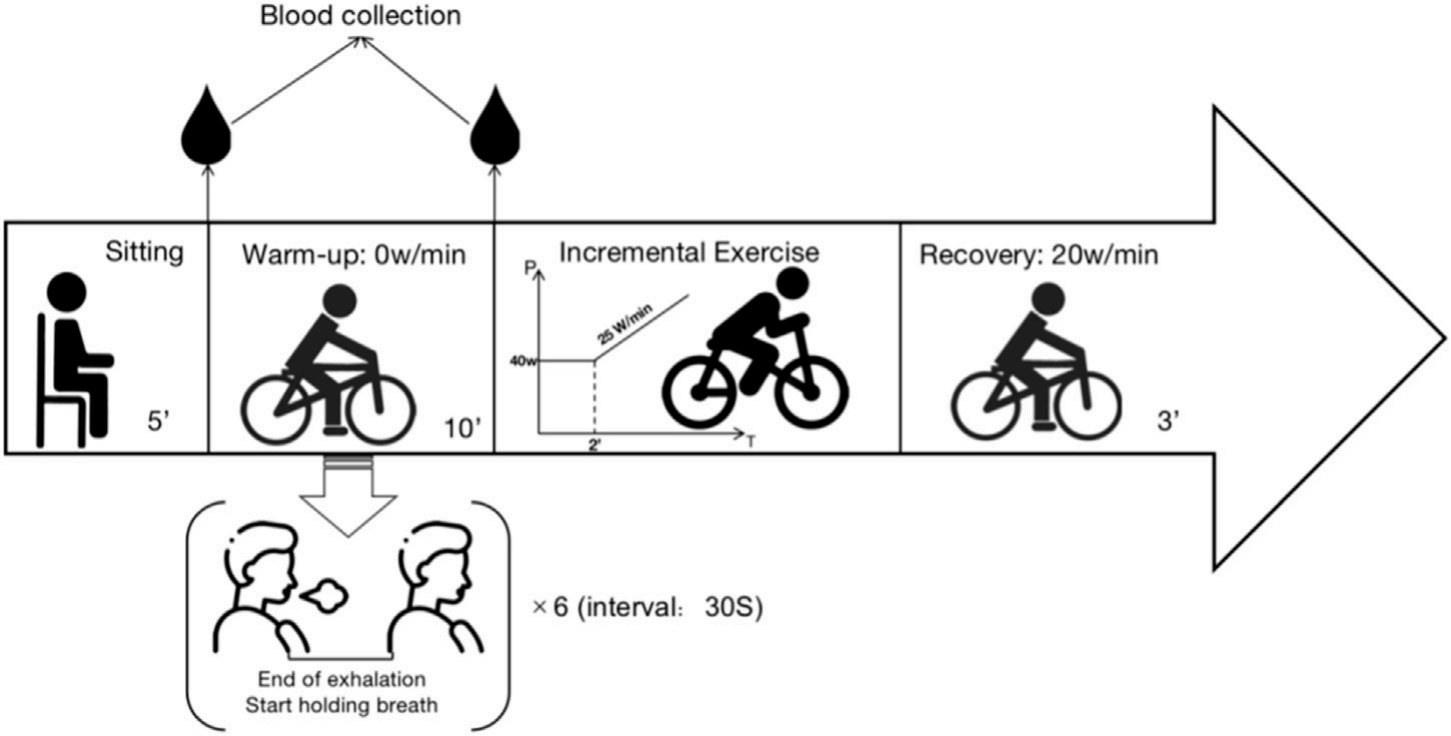
Experiment procedure.

6. Recovery Phase: Participants pedal at 20 W/min for 3 min to facilitate recovery. Throughout the entire test, participants monitor their pedaling speed on the electronic speedometer of the exercise bike, maintaining it between 60–70 r/min. Verbal encouragement is provided by the testing personnel based on the participant’s performance at the point of exhaustion.
7. Conclusion Phase: After the experiment, participants engage in stretching and relaxation for 30–60 min before leaving the laboratory, provided no abnormal conditions are observed. The specific experimental procedure is shown in Figure 2.

### 2.5 Capillary blood collection

The procedure for capillary blood collection (fingertip) adhered to the Chinese Consensus on the Operation of Capillary Blood Collection. It involved disinfecting the puncture point with a 75% ethanol cotton pad and allowing it to dry. The capillary blood collection device was held securely in the subject’s fingers, placed on the skin surface of the subject’s fingers, and the subject was informed of the impending puncture. Following activation, the capillary blood collection device performed the puncture, after which it was removed from the skin and disposed of in a sharps container. The first drop of blood was wiped away using a sterile cotton pad to prevent interference with body fluids. The first and second blood collections were done to ensure that no repeat punctures occurred at the same site (i.e., two punctures). Blood samples were immediately transferred to the laboratory to measure various blood parameters, including RBC, hemoglobin (HGB), hematocrit (HCT), mean corpuscular volume (MCV), mean corpuscular hemoglobin (MCH), and mean corpuscular hemoglobin concentration (MCHC).

### 2.6 Security monitoring

HR was continuously recorded using a Polar HR monitor (Polar Electro, Kempele, Finland) throughout the test. The test was halted when any three of the following conditions were met (Wang, 2015): (1) HR exceeded 180 bpm or failed to rise within 2 min; (2) respiratory quotient was equal to or greater than 1.15; (3) the incremental oxygen uptake between two consecutive sessions was less than 2 mL/(kg·min) or decreased. During breath-holding, subjects were strictly observed for signs of hypoperfusion, such as changes in the color of lips, fingertips, cold sweat, or dizziness.

### 2.7 Subject education

One week before the baseline test, experimenters conducted group training for the subjects, which mainly included the following components: (1) Introduction of breath-holding-related knowledge to enhance the subjects’ foundational understanding and interest in breath-holding; (2) Familiarization of the subjects with the laboratory environment, including the cardiopulmonary exercise laboratory and the entire test procedure; (3) Instruction in breath-holding techniques, emphasizing the importance of avoiding excessive inhalation, swallowing movements, and exhalation during breath-holding.

### 2.8 Data analysis

Peak oxygen uptake (VO2peak) was defined as the highest 30-s average. Peak power output (Ppeak) and HRpeak were defined as the power and HR corresponding to VO2 peak. HRmax was determined as the highest heart rate reached during the incremental exercise test. COP was defined as the point with the lowest ventilation ratio per minute to oxygen uptake per minute during incremental exercise. The measurement of dynamic breath-holding times involved using a timer, with the average value representing the apnea performance.

### 2.9 Statistical analysis

Statistical analyses were performed with IBM SPSS(Statistical Product and Service Solutions)26.0. All data were presented as mean ± standard error. The cardiopulmonary and hematological parameters before and after warm-up were tested for normality by the Shapiro-Wilk test first. Paired samples *t*-test was performed for indicators that conformed to the normal distribution. Wilcoxon rank test was used for the paired data that did not follow the normal distribution. The Pearson correlation test was used to test the correlation between the average breath-holding time and other indicators. Statistical significance was set at *p* < 0.05.

## 3 Results

All subjects had no adverse reactions during the experiment. The baseline warm-up group recorded an RPE result of 18.8 ± 1, while the DA warm-up group yielded a result of 19 ± 0.9, showing no statistically significant difference between them (*p* = 0.616).

### 3.1 Immediate exercise effect

**FIGURE 3:**
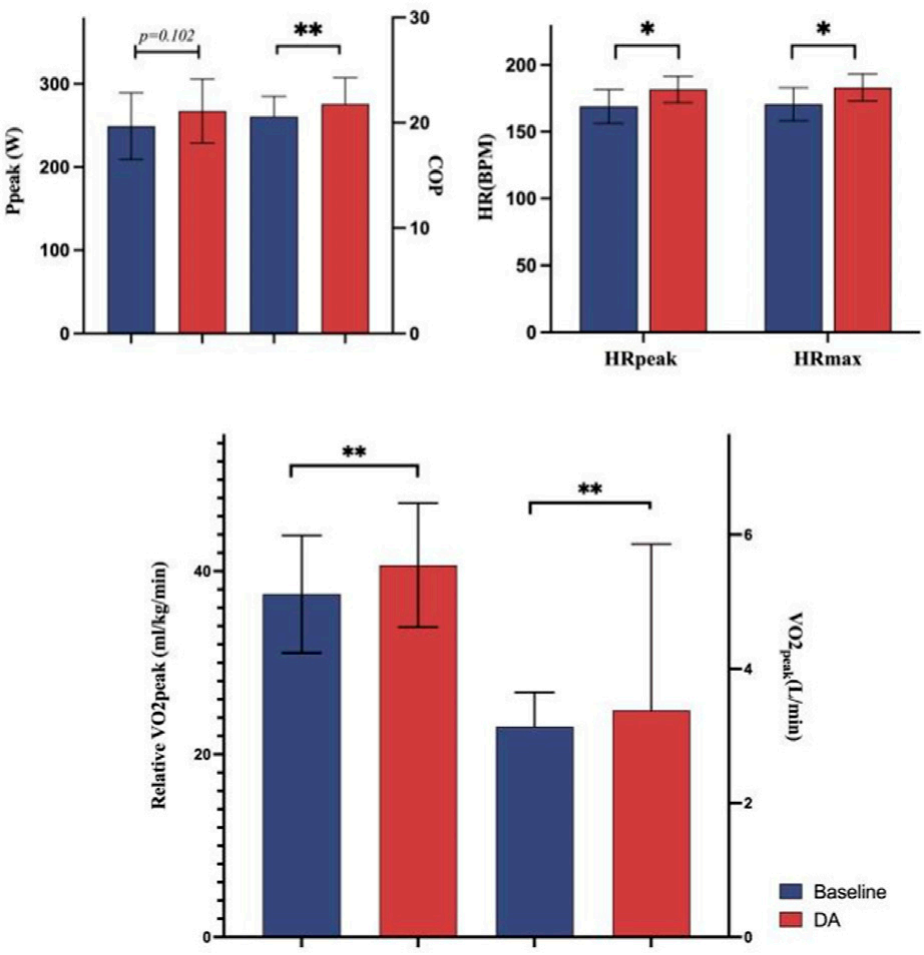
Comparison of CPET—related indicators.

In comparison to the baseline warm-up, several indicators exhibited marked improvement, except Ppeak. Specifically, following the DA warm-up, compared to the baseline warm-up group, COP increased by 5.87%, VO2peak by 7.64%, relative VO2peak by 8.42%, HRpeak by 7.52%, and HRmax by 7.34%. Detailed information on these results is presented in Figure 3.

Specific 95% confidence intervals and ranges (minimum and maximum values) for all indicators can be found in Table 2.

**TABLE 2:** CPET-related parameters.

	Minimum(B)	Maximum(B)	95% CI (B)	Minimum (DA)	Maximum (DA)	95% CI (DA)
COP	17.4	24.56	(20.3, 22.0)	17.68	26.07	(20.6, 22.9)
VO2peak (L/min)	2.22	4.25	(2.8, 3.4)	2.37	4.58	(3.3, 3.8)
VO2peak (mL/kg/min)	27.64	52.48	(33.2, 40.7)	25.08	57.57	(37.1, 45.4)
Ppeak (w)	170.99	318.72	(226.9, 267.1)	179	319	(248.9, 286.0)
HRpeak (bpm)	134	186	(164.5, 177.6)	108	184	(178.6, 192.4)
HRmax (bpm)	132.1	182	(162.5, 174.5)	173.2	213.2	(190.6, 186)

Note: B, Baseline; DA, dynamic apnea; 95% CI, 95% confidence interval.

### 3.2 Hematological parameters

#### 3.2.1 Sample homogeneity test

Hematological parameters can be influenced by external environmental factors over a short duration. To assess whether significant external disturbances occurred between test days, despite a 1-week gap between them, we conducted a homogeneity test on the pre-test indicators of the first and second test days. Statistical analysis revealed no statistically significant differences (*p* > 0.05) in any of the hematological parameters, as displayed in Table 3.

**TABLE 3:** Sample homogeneity test.

Hematological parameters	Pre(B)	Pre(DA)	*P*
RBC (10^12^/L)	5.14 ± 0.89	4.76 ± 0.71	0.16^a^
HCT (%)	45.86 ± 7.42	43.13 ± 17.31	0.48^a^
HGB (g/L)	158.88 ± 26.38	145.62 ± 19.83	0.07^a^
MCV(fl)	89.93 ± 4.35	87.28 ± 2.27	0.06^a^
MCH(pg)	31.36 ± 1.79	30.57 ± 2.27	0.06^a^
MCHC(g/L)	348.73 ± 11.83	350.46 ± 10.11	0.84^a^

Note: RBC, red blood cell count; HGB, hemoglobin; HCT, hematocrit; MCV, mean corpuscular volume; MCH, mean corpuscular hemoglobin; MCHC, mean corpuscular hemoglobin concentration.

^a^Paired samples *t*-test; B, Baseline; DA, dynamic apnea.

#### 3.2.2 Warm-up effects on hematological parameters

Following the baseline warm-up, RBC increased from 5.14 ± 0.89 to 5.26 ± 1.19 × 10^12/L, indicating a growth rate of 2.53%; Hematocrit (HCT) rose from 45.86% ± 7.42% to 46.95% ± 9.97%, reflecting a growth rate of 2.37%; Hemoglobin (HGB) levels increased from 158.88 ± 26.38 to 164 ± 33.54, with a growth rate of 3.22%. After DA warm-up, RBC increased from 4.76 ± 0.71 to 4.85 ± 0.78, representing a growth rate of 1.92%; HCT increased from 43.13 ± 17.31 to 45.26 ± 12.93, showing a growth rate of 4.96%; HGB increased from 145.62 ± 19.83 to 148.19 ± 22.95, indicating a growth rate of 1.77%. These changes did not reach statistical significance (*p* > 0.05). Further details are provided in Table 4.

**TABLE 4:** Differences in hematologic changes.

Hematological parameters	Pre	Post	*P*	Growth Rate(%)
RBC (10^12^/L)				
B	5.14 ± 0.89	5.26 ± 1.19	0.987^a^	2.53
DA	4.76 ± 0.71	4.85 ± 0.78	0.948^b^	1.92
HCT (%)				
B	45.86 ± 7.42	46.95 ± 9.97	0.941^a^	2.37
DA	43.13 ± 17.31	45.26 ± 12.93	0.943^b^	4.96
HGB (g/L)				
B	158.88 ± 26.38	164 ± 33.54	0.973^a^	3.22
DA	145.62 ± 19.83	148.19 ± 22.95	0.862^b^	1.77
MCV(fl)				
B	89.93 ± 4.35	89.85 ± 4.36	0.426^b^	−0.09
DA	87.28 ± 2.27	87.78 ± 2.99	0.426^b^	0.57
MCH(pg)				
B	31.36 ± 1.79	31.34 ± 1.98	0.726^a^	−0.06
DA	30.57 ± 2.27	30.63 ± 1.29	0.815^b^	0.20
MCHC(g/L)				
B	348.73 ± 11.83	348.67 ± 12.96	0.31^a^	−0.02
DA	350.46 ± 10.11	348.87 ± 11.24	0.172^a^	−0.45

Note: RBC, red blood cell; HGB, hemoglobin; HCT, hematocrit; MCV, mean corpuscular volume; MCH, mean corpuscular hemoglobin; MCHC, mean corpuscular hemoglobin concentration.

Baseline% = [Post-warmup (Baseline)-Pre-warmup (Baseline)]/Pre-warmup (Baseline) * 100%.

DA% = [Post-warmup (DA)-Pre-warmup (DA)]/Pre-warmup (DA) * 100%.

^a^Paired samples *t*-test.

^b^Wilcoxon test.

B, Baseline warm-up; DA, Dynamic apnea warm-up.

### 3.3 Breath-holding time

**FIGURE 4:**
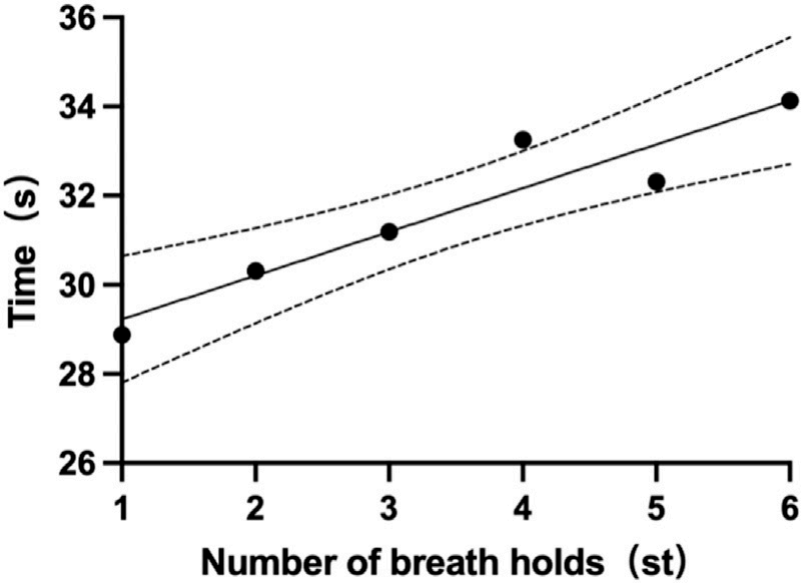
Mean single breath-holding time.

Each participant had six single breath-holding time (BHT). The mean BHT of all subjects is 31.7 ± 12.5s (one subject’s data was missing). The shortest mean BHT recorded was 14.7s (subject 14), while the longest mean BHT was 54.5s (subject 4). Specifics of BHT can be found in Table 5. Changes from the first to the sixth single BHT are shown in Figure 4.

**TABLE 5:** Breath-holding time (BHT).

Subject number	1st	2nd	3rd	4th	5th	6th	Mean BHT(s)
1	30	36	35	36	35	34	34.33
2	24	29	28	29	39	40	31.50
3	20	25	25	30	32	33	27.50
4	40	45	53	58	67	64	54.50
5	27	30	25	33	28	24	27.83
6	30	58	42	60	51	55	49.33
7	20	18	29	30	30	26	25.50
8	78	53	69	45	20	44	51.50
9	45	17	18	20	25	22	24.50
10	20	20	15	21	24	15	19.17
11	18	26	21	20	24	21	21.67
12	20	24	25	34	31	28	27.00
13	20	19	20	21	12	19	18.50
14	12	17	15	14	12	18	14.67
15	40	38	42	46	57	64	47.83
16	18	30	37	35	30	39	31.50
Mean Single BHT(s)	28.88	30.31	31.19	33.25	32.31	34.13	31.7 ± 12.5

Note: 1∼6th: Maximum breath holding time for each subject from the first to the sixth time.

In addition, our study revealed a statistically significant moderate positive correlation between Mean BHT and HRpeak (r = 0.634), HRmax (r = 0.613), and a significant moderate negative correlation with VO2peak (r = −0.547). Detailed correlation results are presented in Table 6 below.

**TABLE 6:** Breath hold time correlation.

	HR_peak_ (bpm)	HR_max_ (bpm)	VO_2peak_ (L/min)	Relative VO_2peak_ (mL/kg/min)	RBC (10^12^/L)	HGB (g/L)
Correlation coefficient	0.634*	0.613*	−0.547*	−0.18	0.313	0.344
*P*	0.02	0.02	0.04	0.54	0.28	0.23

Note: * for *p* < 0.05; VO2peak, Peak oxygen uptake; Relative VO2peak, Relative peak oxygen uptake; HRpeak, Peak heart rate; HRmax, Maximum heart rate; RBC, red blood cell; HGB, hemoglobin.

## 4 Discussion

Enhancing peak performance poses a significant challenge for elite athletes who are already in exceptional physical condition. Even a small increase in aerobic power can have a significant impact. Commencing with a warm-up can boost blood flow and prepare the body for exercise, ultimately leading to improved performance. Past studies have shown that DA can increase athletes’ maximum oxygen uptake, and this effect lasts for up to 10 min after breath-holding (Schagatay et al., 2005; Lemaître et al., 2009). Benefits of breath-holding at low lung volume (referred to as ‘end-expiration’ breath-hold (Woorons, 2014)) include causing significant arterial oxygen desaturation (Woorons et al., 2021), muscular and cerebral deoxygenation (Woorons et al., 2010), and a combination of hypoxic and hypercapnic effects during exercise (da Rosa, 2022). Therefore, this study employed end-expiratory breath-holding and lower extremity movement as a warm-up for DA.

This study delves into the efficacy of utilizing DA as a warm-up technique for enhancing immediate aerobic power, encompassing both immediate exercise power and alterations in hematological parameters. Furthermore, we have also probed into potential correlations between BHT and pertinent influencing factors.

### 4.1 Immediate effects on aerobic power

Aerobic power serves as a key indicator reflecting cardiorespiratory function and aerobic power. There is considerable value in observing the effects of aerobic training and developing scientific training programs (Poole and Jones, 2017). Additionally, breath-hold divers exemplify individuals with heightened aerobic power, attributed to factors such as improved oxygen and CO2 storage and enhanced oxygen-conserving mechanisms (Liner and linnarsson, 1994; Costalat et al., 2015). Additionally, their responsiveness to changes in breathing is diminished compared to non-diving individuals (Roecker et al., 2014). Studies have shown that a decrease in maximal cardiac output leads directly to a decrease in VO2PEAK, suggesting that single intervention means may be an influential factor in influencing immediate VO2PEAK(Al-Samir et al., 2016).

Our hypothesis revolves around the potential influence of a single pre-competition intervention on subsequent aerobic power. The results of this study indicate that the VO2Peak measured with a DA warm-up is significantly higher than the corresponding results obtained with a non-DA warm-up, and this difference is statistically significant. Several plausible mechanisms may contribute to this phenomenon. Notably, breath-holding immediately diminishes the activity of pulmonary tract receptors, reducing vagus nerve stimulation and attenuating the inhibition of inhalation caused by the pulmonary tract reflex (Undem and Nassentein, 2008; Mazzone and Undem, 2016). This reduction potentially enhances oxygen uptake. Moreover, inhibiting inspiration during the warm-up phase may activate feed-forward mechanisms, leading to a reflexive increase in inspiratory volume during the exercise phase (Whipp and Ward, 1982; Woorons et al., 2017). In essence, these findings underscore the positive impact of breath-hold warm-up on VO2Peak, shedding light on its potential role in optimizing aerobic performance.

COP is a submaximal measure reflecting the interplay between cardiovascular and respiratory systems, assessed by VO2 efficiency (Ramos et al., 2012). For majority of healthy individuals, the anaerobic threshold lies at exercise intensities between 50% and 75% of VO2max, while in trained endurance athletes, it can reach intensities as high as 80% of VO2max (Balady et al., 2010). This value signifies the minimum ventilation required to extract 1 L of oxygen, indicating the efficiency of respiratory oxygen consumption during exercise (Ramos et al., 2012; Ramos and Araújo, 2017; Silva et al., 2018). Our study revealed that the COP measured after the DA warm-up was slightly higher than that measured after the baseline warm-up. This variation could be associated with the following mechanism: the discrepancy in COP is primarily explained by CO2exp at VO2max and VE at VT2. The subsequent accumulation of CO2 due to multiple successive maximal breath-holds serves as a source of stimulation for subsequent respiration (Bannister et al., 1954). Past research has demonstrated that sympathetic activity increases expiratory α motor neuron firing in an enhanced manner (Bainton et al., 1985). Breskovic et al. (2011) noted that muscle sympathetic nerve activity continues to increase linearly at the end of breath-hold, suggesting post-breath-hold sympathetic activation. In essence, sympathetic activation after DA leads to increased ventilation during subsequent exercise, resulting in an elevation of COP. It's crucial to note that the rise in COP in this context should not be equated with a decrease in VO2 efficiency. Previous studies have typically considered COP as an indicator of VO2 efficiency after long-term training. However, this study involves a single intervention, and the increase in COP can be considered an immediate change in respiratory metabolism.

Furthermore DA warm-up increased HRmax and HRpeak by 7.34% and 7.52%, respectively, in comparison to the baseline warm-up (*p* < 0.05). Costalat et al. (2015)study identified three key stages during breath-holding: an early phase, a normoxic phase, and a hypoxic phase. In the early phase, HR exhibited an exponential decrease, followed by a slight linear rise, and subsequently, another decrease in HR correlated with a significant drop in oxygen saturation. The third phase in the model presented a linear decrease in HR, featuring unique characteristics distinct from the first two phases. In this stage, HR exhibited a linear decrease, possibly accompanied by further enhancement of parasympathetic activity, indicating a further reduction in the cardiac workload during breath-holding. This phase is immediately followed by subsequent exercise, potentially contributing to a further reduction in the intensity of cardiac activity, thereby achieving more efficient oxygen utilization. It is suggested that activating this mechanism may enhance oxygen preservation capacity during subsequent exercise. Furthermore, Kiviniemi et al. (2012) identified stronger sympathetic activation during DA. This association may imply that central instructions about movement can elicit vagus nerve withdrawal with a sustained effect (Tulppo et al., 1996). As a warm-up technique, it is hypothesized that DA may advance the activation of cardiac sympathetic nerves through a similar feed-forward mechanism, modulating autonomic balance and promoting sustained sympathetic activation during subsequent exercise (Fagius and Sundolf, 1986; Leuenberger et al., 2001; Dempsey et al., 2002). This, in turn, enables the body to respond more swiftly and robustly to the heightened oxygen demands of high-intensity exercise by elevating HR, thereby enhancing immediate exercise capacity. However, our study did not observe any bradycardia effects associated with apnea that could potentially mask exercise-related tachycardia, which might be linked to exercise intensity.

### 4.2 Immediate effects on hematological parameters

Exposure to hypoxic conditions stimulates an increase in RBC and HGB, both pivotal factors influencing endurance capacity (Buick et al., 1980; Kanstrup and Ekblom, 1984; Berglund, 1992). Long-term DA presents itself as a potentially effective method for augmenting erythropoietin (EPO), RBC, and HGB (Levine et al., 1997; Engan et al., 2013). Our study, however, found no statistically significant difference in RBC and HGB before and after warm-up (*p* > 0.05). It's noteworthy that the growth rate of RBC and HGB during baseline warm-up exceeded that observed during DA warm-up.

This incongruity led us to investigate two potential mechanisms. First, as discussed earlier, the intensified spleen contraction under hypoxic conditions could facilitate the release of stored RBC into the circulatory system, potentially raising the levels of RBC and HGB in the bloodstream (Woorons et al., 2010; Woorons et al., 2016; Woorons et al., 2019). This seems to contrast with the findings of our study. Second, research has indicated that blood factors may decrease during exercise, potentially impacting DA warm-ups. As blood oxygen levels drop, EPO levels increase in a dose-dependent manner (Cokic et al., 2014). Various training methods, including hypoxic training known as “live high, train low” (Tee et al., 2023), offer means to stimulate hypoxia effectively. A study by Bruijn (2008) demonstrated a 16% increase in EPO concentration within 3 h after repeated breath-holding (Buick et al., 1980). DA is a similar means of immediate intervention to organismal hypoxia. Nevertheless, our study did not measure blood factor concentrations, necessitating further investigation.

Additionally, our study’s results diverge from prior research (Nazarali et al., 2012), which reported an increase in RBC and HGB after warm-up. This difference may be attributed to the involvement of elite athletes with higher aerobic-related blood parameters in our study. Individuals who do not regularly engage in exercise may experience more noticeable spleen contractions (Holmström et al., 2021).

When comparing the growth rates of HCT, we observed that the HCT growth rate during DA warm-up exceeded the baseline rate. HCT reflects the proportion of RBC in the blood volume and is influenced by both RBC and plasma volume (Richardson et al., 2005). Given the absence of a significant change in RBC and HGB in our study, we speculate that the increased HCT growth rate may be attributed to a decrease in plasma volume. Temporary reductions in plasma volume of up to 6%–20% have been reported during strenuous exercise (Andersson and Schagatay, 1998). The rise in the HCT growth rate observed during DA warm-up can be explained by several factors.

First, breath-holding induces the compression of venous small blood vessels, increased blood flow to capillaries, and elevated capillary pressure, resulting in plasma water leakage into the extravascular space (Harrison et al., 1975), thereby increasing blood concentration (Pivarnik et al., 1984). Second, Elia found that DA results in higher lactic acid production compared to static breath-holding training (Elia et al., 2021a). This increase in lactic acid may worsen DA by causing fluid to move from blood vessels to surrounding tissues, leading to a decrease in plasma volume (Kraut and Madias, 2014). This decrease can positively impact aerobic power, warranting further investigation. Moreover, increased perspiration can also lead to a reduction in intravascular plasma volume.

### 4.3 Breath-holding time-related indicators

Breath-Holding Time (BHT) stands as a widely utilized metric in respiratory physiology for evaluating ventilatory responses, and finding applications across health assessments, disease diagnostics, and various domains (Parkes, 2006; Godfrey and Cambell, 1968). In our investigation, the six voluntary BHT measurements averaged 31.7 ± 12.5 s, showcasing a lower figure compared to the average BHT noted in healthy adults, as seen in Fowler, (1954) study. It's crucial to highlight that the breath-hold in our study commenced at the end of exhalation, differing from most prior studies, including Fowler, (1954), which initiated it at the end of inhalation.

In marine mammals, elevated hemoglobin levels are considered an advantageous adaptation for diving (Ponganis, 2011). However, in humans, despite reports of increased red blood cells and hemoglobin in divers (Elia et al., 2021b), a direct correlation between HGB and breath-hold capacity seems elusive. Our study’s results align with conventional wisdom, revealing no significant correlation between BHT and RBC or HGB (*p* > 0.05).

Importantly, our study has identified a significant correlation (*p* < 0.05) between BHT and VO2PEAK, HRmax, and HRPEAK. We observed that a longer BHT was associated with a higher HRmax and a lower VO2PEAK, aligning with previous findings indicating no correlation between breath-holding and activities not requiring maximal effort (Montoye, 1951).

Extensive research has explored factors influencing BHT, revealing its sensitivity to physiological factors like lung or chest constraints (e.g., vital capacity and initial lung volume), mechanisms related to blood gas pressure or carotid chemoreceptors (Mithoefer, 1959a; Delapille et al., 2001; Skow et al., 2015), and actions such as prolonged inhalation, achieved through lung inflation, high oxygen levels, and hypocapnia. Conversely, decreased lung inflation and increased metabolic rates shorten breath-holding duration (Vigran et al., 2019). Smoking habits, body size, and age also influence BHT (Sudha et al., 2012).

Psychological factors and practical experience significantly shape BHT, with self-control, calmness, and anxiety levels inducing notable changes (Kyriakoulis, 2019). Regular breath-holding training gradually enhances lung capacity and increases carbon dioxide tolerance, extending BHT (Mamatha and Gorkal, 2012). However, training effectiveness may vary based on individual adaptations and frequency. Prior research utilized BHT in cystic fibrosis patients to predict physical performance (Barnai et al., 2005). In contrast, our study focused on athletes with no pathological changes in lung capacity, highlighting the potential influence of willpower and physical activity levels.

Our study aimed to minimize the impact of previous respiratory training on BHT, selecting athletes without breath-holding training or experiences in activities like yoga and meditation. However, quantifying the combined effects of individual willpower and CO2 tolerance on BHT may be challenging. The measurement involved oral prompts and observations, introducing potential hand-eye delay and error. Despite a simple correlation analysis yielding significant results, further analysis is challenging due to these limitations, indicating areas for improvement in future research as we strive for a more accurate understanding of the BHT-athlete performance relationship.

In our study’s preliminary experiment, similar conclusions were drawn, indicating that BHT doesn't necessarily correlate with higher peak oxygen consumption. Directly concluding that a longer BHT corresponds to lower peak oxygen consumption proves challenging, considering the limited sample size. Recognizing this, future research should expand the participant pool to mitigate statistical errors.

Future studies could delve deeper into the relationship between DA and aerobic power, considering factors such as lung volume measured through body volume scans (Borg and Thompson, 2012) and physiological indicators like muscle content assessed through body composition tests (Lohman et al., 2019). These measures would provide a more comprehensive understanding of factors influencing BHT and its connection with aerobic exercise capacity, contributing to the development of effective training programs and strategies for athletes.

### 4.4 Innovations

This paper presents three key innovations. First and foremost, it distinguishes itself from prior breath-holding exercises by initiating the dynamic breath-holding phase at the conclusion of a natural expiration, as opposed to starting after maximal inspiration. This departure from convention is grounded in a fundamental understanding of the physiological consequences of breath-holding during inspiration. When breath is held during the inhalation phase, it triggers lung expansion and diaphragmatic contraction, necessitating heightened abdominal muscle contractions to maintain intra-abdominal pressure. This, in turn, results in premature depletion of physical reserves before the commencement of the exercise. Furthermore, it compromises the abdominal core strength required for lower limb pedaling. Consequently, this paper’s approach opts for commencing the warm-up phase at the end of natural expiration to preserve physical strength.

Secondly, a comprehensive review of the literature reveals a substantial disparity in the training methods for dynamic breath-holding, including variations in exhalation pause durations, maximal breath-holding periods, and repeated breath-holding episodes during short rest intervals (Fernandez et al., 2018; Fornasier-Santos et al., 2018). Based on our team’s prior experiments, we have determined that the most suitable warm-up method involves maximal repetitions of voluntary breath-holding. This method effectively mitigates discrepancies arising from differences in individuals’ mental capacities, ensuring a more uniform and controlled warm-up process.

Finally, a body of research has established the crucial role of exhaled nitric oxide (NO) in the regulation of pulmonary vascular tone and the facilitation of oxygen transport. Given NO’s high affinity for hemoglobin, holding one’s breath at the end of exhalation is more likely to diminish endogenous NO concentrations in the alveoli compared to breath-holding at the end of inhalation. This nuanced approach ultimately enhances oxygen consumption and augments aerobic power.

### 4.5 Limitations

In our study’s methodology, we acknowledge some limitations. To allow athletes’ mobility during DA and avoid a venous catheter, we opted for fingertip capillary blood for Hb measurements. However, we recognize potential reliability issues due to the diving reflex affecting finger blood circulation (Neufeld et al., 2002). Despite these considerations, fingertip capillary blood sampling for Hb analysis is commonly used in studies (Holmström et al., 2021; Lodin-Sundstrom et al., 2021). Another limitation is the sample size. While we calculated the effect size and found 17 subjects reasonable, future research could benefit from a larger and more diverse participant pool for better generalizability, given the variability in BHT and physiological responses.

## 5 Conclusion

Overall, this study explores the immediate effects of using DA as a warm-up in top athletes on aerobic power, hematological parameters, and BHT. DA significantly increased aerobic power, which was manifested by higher VO2Peak values, possibly due to decreased pulmonary receptor activity and increased oxygen uptake during exercise. The study found no significant changes in RBC and HGB levels after DA warm-up, but an increased rate of HCT growth was observed, which may be related to changes in plasma volume. Notably, there was a significant correlation between BHT and aerobic power, HRmax, and HRpeak, suggesting a possible link between breath-holding capacity and cardiovascular response during exercise, but further research is needed to understand how these effects perform in different populations and to explore the long-term effects of incorporating DA into pre-competition training.

## Data Availability

The original contributions presented in the study are included in the article/Supplementary Material, further inquiries can be directed to the corresponding authors.
